# Annual Ailments

**Published:** 1881-04

**Authors:** 


### *ANNUAL AILMENTS.*

We eat about one-third more in winter than in summer, because we not only have to repair the wear and waste of the system, but we eat to keep the body warm, a portion of the food is converted into fuel; we must keep a bodily warmth of ninety or a hundred degrees winter and summer, but it is easy to understand, that, as the thermometer is at forty in winter and eighty in summer, less fuel is required to sustain the natural temperature in warm weather; yet, if in defiance of this, we pile on the fuel; a wreck and ruin is as inevitable as the blowing up of a steam engine, if double the necessary quantity of steam is constantly generated.

For a while after the opening of spring, we have the appetite of winter, and not using our knowledge, we indulge it as extensively; and thus generating more heat than is needed, we soon begin to think "we are feverish," in other words, we are too warm, but instead of making less fire, we begin to tear down the walls of our bodily-house, by taking off our winter clothing, and thus add another cause of disease and death. In a short time, however, nature comes to our aid, and to save us, takes away our appetite; but we, taking this as an evidence of declining health, decide upon one of two things: either to eat without an appetite—which is expressively denominated as "*forcing it down*"—or we decide upon taking a tonic, forgetting that nature can neither be forced nor coaxed with impunity. The effect of eating without an appetite, or forcing an appetite by the use of tonics, is the same, that is, the introduction of more food into the stomach than nature requires, than there is juices to digest it; for, although you may take a tonic which whets the appetite, it does no more; it does not increase the amount of gastric juice, for nature supplies it only in proportion to the needs of the system, and if she gave as much when twenty degrees of heat were required as when sixty were necessary, she would commit a great blunder—this she never does, when unmolested. Then we have more food in the stomach than there are gastric juice for; more wheat than there are mills to grind it more work than there are workmen to perform. But nature has not "a lazy bone in her," but goes to work to do the best she can; the food is digested, but not thoroughly; it is ground up, but not perfectly; the work is done, but it is badly done; hence an imperfect material for making blood is furnished; and an impure blood, an imperfect blood, is inevitable. Do not many of us recollect the old time custom of taking "sassafras" tea in spring, or some other favorite remedy, with the expressed intention of "purifying the blood?" It is a habit with multitudes to use some kind of medicine in the spring of the year, and beyond all question with present good effect, as they all act in one way essentially, and that is, to remove the surplus from the system. It all amounts to this: we eat in the spring more than we can dispose of, and then take medicine to get rid of it, and all for the transient pleasure enjoyed for the few minutes of each day that it is passing down the throat. But some are "principled," as they term it, against taking physic; but they are not "principled" against the greater harm of eating against the appetite, for by taking the physic they would be consuming something which might destroy others; whereas, by eating a meal without an appetite, they consume—and that to their own injury—that which would save many a famishing creature, man or beast, from starvation. None can read without disgust of a Roman Ruler, who would eat to his full, then take an emetic, that he might eat again, or be saved from the effects of a gorge. Even this person acted more wisely than does he who eats without an appetite, and allows it to remain in him to vitiate the blood and finally destroy the body, but in the slow process of destruction, affording time to transmit to the innocent unborn, a vitiated constitution, to afflict and plague for untold years to come. If the man could but die in the act, as it were, the world would be left the better, for there would be more left to be eaten by the more worthy, and he would not leave his slimy trail behind him, in the person of a child. If he is said to be a real benefactor to his race who makes two blades of grass grow where but one grew before, what ought he to be thought of, who absolutely destroys food each day by forcing it down, which it would take a million blades of grass to reproduce? Reader! do you plead guilty to having eaten without an appetite, to having "forced it down?"

then do it no more, for it is a sin against yourself, against nature, and against all human kind.

We are all familiar with the prevalence of bowel-complaints of all kinds, in the spring of the year, and of their fatal nature, sometimes spreading from house to house, from family to family, from neighborhood to neighborhood, like some infectious or contagious disease, and often, but most erroneously, attributed to the use of fruits, berries, and the like; the cause is one and universal: it is over eating, with its legitimate results, sour stomach, wind, loose bowels, debility, diarrhœa, dysentery, and death. Thus it is, that the more sudden the coming on of spring weather, and the hotter it is, the more sickness there will be, while in the fall of the year, as the weather gets colder, and however suddenly, we begin at once to gain in appetite, in vigor, in flesh and health.

The remedy for spring diseases, by whatever name, is EAT LESS. We do not mean that you shall starve yourself, or that you shall deny yourself whatever you like best, for as a general rule, what you like best, is best for you; you need not abandon the use of tea, or coffee, or meat, or anything else you like, but simply eat less of them. Eat all you did in winter, if you like, but take less in amount. Do not starve yourself, do not reduce the quantity of food to an amount which would scarcely "keep a chicken alive," but make a beginning, by not going to the table at all, unless you feel hungry; for if you once get there, you will begin to taste this and that and the other, by virtue of vinegar, or mustard, or syrup, or cake, or "something nice;" thus a fictitious appetite is waked up, and before you know it, you have eaten a hearty meal, to your own surprise, and perhaps that, or something else, of those at table with you.

The second step towards the effectual prevention of all spring diseases, summer-complaints, and the like, is: diminish the amount of food consumed at each meal by one-fourth of each article, and to be practical, it is necessary to be specific; if you have taken two cups of coffee, or tea, at a meal, take a cup and a-half; if you have taken two biscuits, or slices of bread, take one and a-half; if you have taken two spoonfuls of rice, or hominy, or cracked wheat, or grits, or farina, take one and a-half; if you have taken a certain, or uncertain quantity of meat, diminish it by a quarter, and keep on diminishing in proportion as the weather becomes warmer, until you arrive at the points of safety and health, and they are two:—

1. Until you have no one unpleasant feeling of any kind after your meals.

2. Until you have not eaten so much at one meal, but that when the next comes, you shall feel decidedly hungry.

If these suggestions are attended to in any community, in any spring, the physician in that locality will "book" but twenty-five cents in the dollar. Think, for a moment, how beautiful, and wise, and kind, is nature's mode of procedure in such cases. You may "force" your food for a-while, but at length she goads you on until you loathe its very smell or sight, or even mention. And when some mistaken mother, or sister, or aunt, or granny, prepares you "something nice," and before you are aware of it places it right under your nose, with the assurance that you must take something to keep up your strength, you can only smother your impatience, or hold in your imprecations of left-handed blessings, at the expense of the most heroic efforts. For days and weeks you do nothing but "sit around;" you eat nothing, you loaf and lounge about, more dead than alive, with a countenance nineteen yards long, and so unfeignedly solemn to behold, as to excite the commiseration of the kind-hearted, or the cachination of the healthy.

Supplies being thus effectually cut off, that is, the cause being first removed, nature next proceeds to work off the surplus, as the engineer does unwanted steam; and as soon as this surplus is got rid of, we begin to improve; the appetite, the strength, the health return by slow and safe degrees, and we at length declare we are "as well as ever."

Now, if instead of eating against nature, we would do at once what nature will enevitably compel us to do, sooner or later, that is, lessen supplies, at the very first and faintest intimation of approaching ill, we would, *if done under the counsel of a regular physician*, not lose an hour from our daily avocations, and would be as well as ever at the end of a week, instead of at the end of months, and save, too, all the months of suffering, and making them, as to pleasure or business, the blanks of our existence.

